# A Gamification-Based Intervention Program that Encourages Physical Activity Improves Cardiorespiratory Fitness of College Students: ‘The Matrix rEFvolution Program’

**DOI:** 10.3390/ijerph17030877

**Published:** 2020-01-30

**Authors:** Jose Mora-Gonzalez, Isaac J. Pérez-López, Irene Esteban-Cornejo, Manuel Delgado-Fernández

**Affiliations:** 1 PROFITH “PROmoting FITness and Health through physical activity” Research Group, Sport and Health University Research Institute (iMUDS), Department of Physical Education and Sports, Faculty of Sports Science, University of Granada, 18071 Granada, Spain; ireneesteban@ugr.es; 2 Educación física y transformación social, SEJ546 Research Group, Department of Physical Education and Sports, Faculty of Sports Science, University of Granada, 18071 Granada, Spain; isaacj@ugr.es; 3 PA-HELP “Physical Activity for Health Promotion, CTS-1018” Research Group, Department of Physical Education and Sports, Faculty of Sports Science, University of Granada, 18071 Granada, Spain; manueldf@ugr.es

**Keywords:** aerobic fitness, health, innovation, university, young adults

## Abstract

The aim of the present study was to examine the effects of a gamification-based program on cardiorespiratory fitness (CRF) levels of college students. We divided 112 college students into an intervention group (IG) and a control group (CG). IG college students followed a 15-week gamification-based program, whereas CG followed traditional lectures. CRF was assessed using the 20-meter shuttle-run test. CRF significantly improved after the program in the IG compared to CG (d ≤ 0.94, *p* < 0.001). Only participants of IG had significant CRF improvements (d ≤ 0.87, *p* < 0.001) between pre- and post-assessments. In the IG, from the students who attended 100% of lectures, 87.8% met physical activity recommendations for 100% of weeks, whereas from those who attended <100%, only 26.7% met them them for 100% of weeks (*p* < 0.001). Participants who met recommendations 100% of weeks had a significant CRF improvement (*p* < 0.001). Motivating college students throughout innovative teaching methods (e.g., gamification) can lead to health improvements.

## 1. Introduction

The World Health Organization recommends at least 150 min of moderate physical activity (PA) per week, 75 min of vigorous PA or an equivalent amount for adults aged 18–64 years [1]. However, adults do not reach public health guidelines for recommended levels of PA and, therefore, PA levels have shown marked declines across adulthood [2,3]. Reduction in PA is usually accompanied by a reduction of physical fitness levels, which may determine present and future health diseases [4,5,6].

The physical fitness components which have been shown with potential to improve health are cardiorespiratory fitness (CRF), muscular fitness and motor ability [6,7]. Namely, there is strong evidence supporting that CRF is the strongest marker of health in young people [6]. In fact, high levels of CRF have been associated with reductions of all-cause mortality and cardiovascular and coronary heart diseases [8]. Novel approaches to promote PA in an effort to improve CRF in young adults are needed. A potential way of influencing young people to engage in PA daily patterns is through the implementation of healthy lifestyle education programs in university settings [9,10]. In this context, college students are considered as a target population since they are one of the most susceptible age-group in acquiring unhealthy habits due to the university way of life (i.e., long studying times, increment of nightlife, lack of budget, etc.) [11]. However, a lack of interventions in university settings for promoting healthy lifestyle behaviours among college students has been identified [12].

With respect to the above, giving the teaching methodologies a gamification approach in universities might be of help to increase motivation of college students and, therefore, to promote and develop PA patterns. Gamification is considered as a motivational tool based on the application of game-design elements and game principles in non-game contexts [13]. Despite the fact that gamification might be a key technique when implementing healthy lifestyle education programs, there is still lack of comprehension on how it works and on how experiences can be designed to achieve health and education improvements [14].

To the best of our knowledge, no studies have analysed the influence of a gamification-based teaching innovation program on CRF of college students. Thus, we implemented a 15-week gamification-based intervention program called “The Matrix rEFvolution” to encourage the practice of PA in college students. Hence, the aim of the present study was to examine the effects of a 15-week gamification-based teaching innovation program on CRF levels of college students.

## 2. Materials and Methods 

### 2.1. Study Design and Participants

A quasi-experimental non-randomized controlled trial with two arms (i.e., intervention group, IG and control group, CG) was carried out from March to June during 2015/2016 academic year. Participants belonging to the IG were college students who, previous to the beginning of the study, had enrolled in one of the three student groups of the subject “Basis of Physical Education and Sport”. This subject belongs to the degree in Physical Activity and Sport Sciences (Faculty of Sport Sciences, University of Granada, Spain). Participants of the CG were also college students from the same subject and degree but not enrolled in the same student group. The selection of a student group was undertaken by the students depending on their schedule preferences.

A total convenience sample of 112 college students (20.93 ± 1.32 years old; 67.9% boys) participated in this study. Of the total sample, 56 were college students (64% boys) belonging to the IG. The CG was also formed by 56 college students (71.4% boys) who did not receive the mentioned gamification-based program and followed instead a traditional teaching methodology (i.e., theoretical and magisterial presentations by the professor and class work by the students oriented to the development of an annual program in physical education). A comprehensive verbal description of the purpose of the study was given to all participants and written consent was requested from them. The study protocol was approved by the Review Committee for Research Involving Human Subjects at the University of Granada (approval number: 421/CEIH/2017).

### 2.2. Procedure

All participants completed a CRF test (see below) at baseline (March, 2016) and post-intervention (June, 2016). After the baseline, the gamification-based PA intervention program started, with a duration of 15 weeks, from 2 March to 17 June 2016. In short, both IG and CG received the same subjects’ content (i.e., basis of physical education, innovation in physical education and programming in physical education). However, the way college students of each group were given the contents differed. Whereas traditional lectures not focused on encouraging PA but instead in giving theoretical contents by the professor were given to the CG, a gamification approach of the subjects’ contents focused on encouraging PA by meeting PA recommendations was followed by the IG. Two different lectures were selected by the Department of Physical and Sports Education to lead each group.

### 2.3. Matrix rEFvolution: A Gamification-Based Physical Activity (PA) Program

A gamification-based program focused on encouraging PA practice was implemented by an experienced lecturer at University of Granada. The program was named “Matrix rEFvolution” (“EF” comes from the Spanish expression “Educación Física”, i.e., “Physical Education”) and consisted of a gamification-based learning experience set in the “The Matrix Revolutions” science fiction film. The program consisted of 42 gamification-based lectures and the gamification technique [13] was used as a motivational teaching tool to encourage college students to overcome a set of learning challenges and to meet PA recommendations the highest possible number of weeks.

For the gamification-based experience, the students were given the role of “rebels” (as in the original film, people of Sion) and belonged to “The Resistance” against Matrix (i.e., corrupt system). The main goal of the students was to become “The Chosen Ones” for what they have to use their potential as “educative hacker” to free the minds of all those teachers felt prey to Matrix. All students participating in the “Matrix rEFvolution” project had a badge to be identified as people of Sion and the lecturer also had a badge to be identified as Morfeo (one of the main characters of the film against Matrix). To be able to overcome the main goal of the adventure (destroy Matrix) from the educative perspective, the students had to train and learn conveniently as the only way to locate the CPU (Central Processing Unit of Matrix that represented the Comfort, Pessimism and Uniformity of other professors) and insert there new software (i.e., represented by innovation teaching project designed and presented by the students) to restart the System and recover the hope and compromise among them.

**Figure 1:**
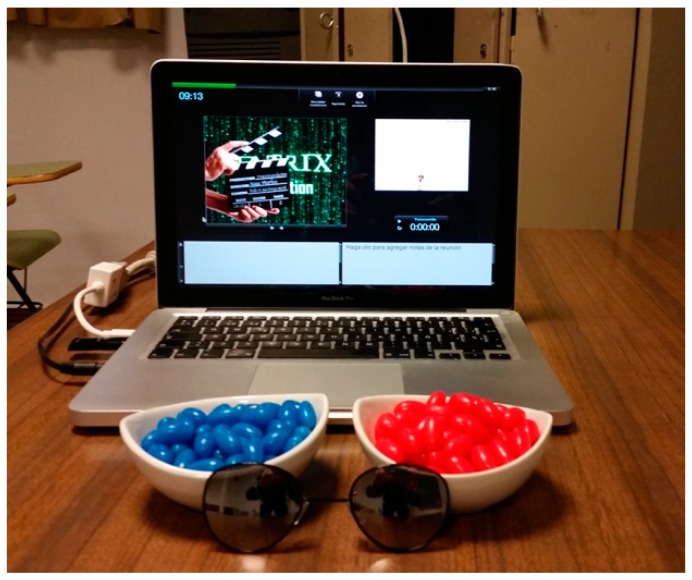
Image of the pills that determine the decision of the students.

One of the main aspects when using gamification in an educative context and a film as a reference is to remain faithful to the film’s script. This helps to increase the credibility of the created adventure and encourage the students’ motivation. An example of being faithful to the script was that experienced by the students the first day of the adventure. Thus, the first thing the students had to do was to discover where Morfeo (the professor) was hiding. For this purpose, they received a call from the professor who asked them to look for the White rabbit. The White rabbit was a QR (Quick Response) code which redirected them to the position of Trinity (represented by a former student) that would be the person who helped them find Morfeo, as happens in the original film. Once they reached Morfeo, he presented the project to the students and asked them to make a crucial choice (as Neo, another character of Matrix, had to do in the film): to choose between taking the blue pill (i.e., to turn their backs on the reality, and choose the comfort) or the red pill (i.e., to face the reality of the education, adopting a critical perspective, and working for a better world) (Figure 1). Therefore, all participants from the IG chose the red pill. 

The subject (i.e., the “Matrix rEFvolution” experience) consisted of 5 different phases taken from the original film: 1. Recruitment; 2. Rehabilitation; 3. Training; 4. Incursion in Matrix; 5. Final Mission. Each of the phases included one or several learning missions designed from the objectives and contents of the subject. A brief description of each phase can be found in Table 1. 

So far, the description of the “Matrix rEFvolution” program has been made from the educative and learning perspective. However, the present study focuses more on the intervention carried out on the PA patterns of the students. This intervention was also established under the framework of “Matrix” and consisted in promoting an active lifestyle behaviour by asking the students to run or cycle from 3 to 5 days per week in order to run away from “The Sentinels” (i.e., simulated villains) and avoid their attacks (i.e., to lose learning points). 

The motivational approach of gamification was based on a classification. For scoring, participants had to meet PA recommendation every week and depending on the quantity (i.e., 3 times per week or 4 times per week registered by mobile phone applications) they were awarded with a specific score. They were also awarded depending on the score obtained when overcoming the learning missions explained above. The achievement of becoming a “Chosen One” to take part in “The Resistance” against Matrix depended on the score obtained and, therefore, on the position in the classification. 

In summary, for the present study the gamification-based intervention program consisted of encouraging college student to meet weekly the internationally accepted PA recommendations [1] during a 15-weeks period by doing 2 possible activities, such as running or cycling in order to run away from “The Sentinels”.

### 2.4. Outcomes

#### 2.4.1. Cardiorespiratory Fitness Assessment

The 20-meter shuttle-run test (20mSRT) was used to assess CRF level [15]. Test-retest reliability of the 20mSRT has been previously shown (r = 0.95) in adults [15]. This test required participants to run back and forth between two lines set 20 meters apart. Running pace was determined by an audio signals with an initial velocity of 8.5 km/h, which was increased by 0.5 km/h every minute. The test finished when the participant failed to reach the end lines concurrent with the audio signal on two consecutive occasions or when the participants stopped because of exhaustion. The total number of completed stages was registered and an estimation of maximal oxygen consumption (VO2max, ml/kg/min) was calculated using Léger equation for adults [15].

#### 2.4.2. Attendance (Level of Implication)

For cross-sectional analyses and on the basis of the fact that the attendance to gamification-based lectures was established as voluntary by the professor, the attendance over the total of 42 gamification-based lectures was registered to assess the grade of implication of the participants with respect to the “Matrix rEFvolution” program developed in the IG.

#### 2.4.3. Number of Weeks Meeting PA Recommendations

The number of weeks that participants met the PA recommendations by running or cycling and, likewise, by successfully running away from “The Sentinels” was also registered for cross-sectional analyses over a total of 15 weeks. To check if participants met the PA recommendations they were required to upload their PA activities per week and, for this purpose, they used the mobile applications of Runtastic (www.runtastic.com) or Endomondo (www.Endomondo.com). Both applications are online sports community based on free real-time global positioning system (GPS)-tracking of running, cycling, etc. The validity of GPS-enabled iPhone “app” to record exercise distance has been previously demonstrated [16].

#### 2.4.4. Satisfaction Assessment

The satisfaction level of participants with respect to the “Matrix rEFvolution” program and the subject was assessed using an adhoc question developed by the professor. The question was “What is your grade of satisfaction with the program?” and consisted of a Likert-type scale (range 1–5) with five response options (very poor, poor, average, good, and very good). 

### 2.5. Statistical Analysis

The characteristics of study sample are presented as means and standard deviations. The differences in baseline characteristics between CG and IG groups were analysed by one-way analysis of variance (ANOVA). The intervention effects on CRF level of college students were studied between groups by one-way analysis of covariance (ANCOVA) adjusting by age and sex and within groups by paired *t*-test. Cohen’s effect size statistics (d) as standardised mean differences between groups were also calculated [17]. Cohen’s d values of 0.2, 0.5 and 0.8 were considered small, medium and large effects, respectively. In addition, analysis of covariance (ANCOVA) was performed adjusting by sex and age to examine the differences in CRF improvements (i.e., post-pre CRF assessment) between groups of weeks meeting PA recommendations (i.e., college students who met PA recommendations <100% of weeks of the program vs. college students who met the recommendation the 100% of weeks of the program) in the IG (n = 56). A significance level of *p* < 0.050 was set. All the statistical procedures were performed using the SPSS software for Windows, version 22.0 (IBM Corporation, New York, USA).

## 3. Results

Table 2 presents the baseline characteristics of the study sample as means and standard deviations. There were no significant differences for any of the baseline characteristics between college students of both CG and IG groups (all *p* ≥ 0.379). Consequently, all models were not further adjusted for baseline levels of the outcome studied.

Table 3 shows the frequency of attendance to gamification-based lectures, the frequency of weeks meeting PA recommendations and the grade of satisfaction with respect to the “Matrix rEFvolution” program of all college students belonging to the IG. Thus, 73.2% of college students attended all gamification-based lectures (i.e., 42 lectures), whereas a 26.8% attended to a range from 37 to 41 lectures. In addition, 71.4% of college students met the PA recommendation the 100% of weeks (i.e., 15 weeks), whereas a 28.6% met them in a range from 11 to 14 weeks. Table 3 also shows the level of satisfaction reported by the college students of IG with respect to the “Matrix rEFvolution” gamification-based program. A mean score of 4.9 ± 0.3 was reported by students of this group. 

**Figure 2:**
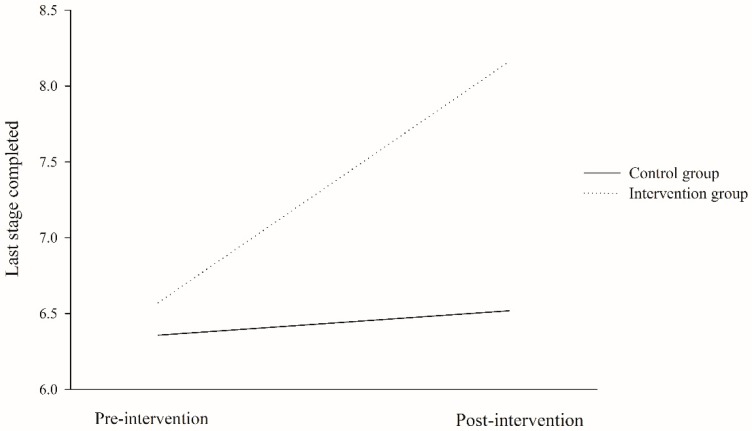
Changes in cardiorespiratory fitness level measured by last stage completed in 20-meters shuttle-run test for intervention and control group (*p* < 0.001, for post-intervention differences between groups).

Pre-intervention, post-intervention and mean differences for CRF level (i.e., last stage completed and estimated VO2max in 20mSRT) between and within groups are shown in Table 4. CRF levels significantly improved after the program in college students from the IG, compared with those from the CG (improvements of 1.7 ± 2.3 stages, d = 0.94; and 5.0 ± 7.3 ml/kg/min, d = 0.93, respectively; all *p* < 0.001). Analyses within group showed no significant differences for CG between pre-intervention and post-intervention assessments neither for stages (d = 0.06, *p* = 0.151) nor for estimated VO2max (d = 0.08, *p* = 0.213), whereas participants of IG had a significant improvement of 1.6 ± 1.0 stages (d=0.86; *p* < 0.001) and 4.8 ± 3.0 ml/kg/min (d = 0.87; *p* < 0.001) between times of assessments. Effects of intervention program on CRF measured by last stage completed were represented graphically in Figure 2. In addition, CRF improved in most of college students belonging to IG (average CRF stages = 85.7%).

Of those college students who attended 100% of lectures, 87.8% of them met the PA recommendations during all weeks of the program. On the other hand, of the college students who attended <100% of lectures, 26.7% of them met the PA recommendations during all weeks of the program. Regarding differences in CRF improvements (i.e., difference post-pre for last stage completed), participants who met PA recommendations the 100% of weeks of the program (i.e., during 15 weeks, every week) had a CRF improvement of 2.0 stages in post-intervention assessment with respect to the pre-intervention, whereas participants who did not met PA recommendations the 100% of weeks only had a CRF improvement of 0.5 stages (*p* < 0.001).

## 4. Discussion

The main finding of the present study suggests that college students participating in a gamification-based program to meet PA recommendations, called “The Matrix rEFvolutions”, improved their CRF levels in comparison with peers from the CG. 

As secondary findings in the IG, we also found significant differences between attending or not to the 100% of gamification-based lectures with respect to meeting or not PA recommendations for 100% of weeks. There were also significant differences in CRF improvements (post-pre stages) between those meeting PA recommendations 100% of weeks and those who did not. These secondary findings could explain the positive ones found when examining the effects of the gamification-based program on IG in comparison with the CG, since a gamification way for leading lectures seems to be powerful for encouraging the practice of PA and, therefore, improving CRF. 

To the best of our knowledge, this is the first study that examines the influence of a gamification approach as a motivational teaching tool to increase PA levels in order to, therefore, improve CRF of college students in a university setting. Despite the fact that there are no studies that have shown the direct influence of gamification on CRF in this education context, there is, however, a growing body of evidence on the effects of PA programs on physical health in general population [18,19,20]. Particularly in adults, a study showed that fast-paced walking of 60–119 minutes per week was associated with clinically meaningful improvements in CRF (mean = 6% increase in VO2max) [21]. Thus, regarding level of PA intensity, higher intensity PA has been linked with higher increments of CRF levels [22]. A randomized controlled trial in adult couples showed significant changes between groups in fitness (*p* = 0.037), as measured by the weight-adjusted physical work capacity at 75%, at the end of a PA program that aimed to achieve at least 30 minutes of moderate PA on most days [23]. Furthermore, the effects of PA programs on CRF have been also shown in a younger population in school settings [18,19]. Taken together, all this evidence regarding the effects of PA on CRF could explain the positive effects of the gamification-based program on meeting the PA recommendations along a 15-week period on CRF levels of college students of the present study. 

A very important factor to take into consideration when programming and adhering PA is motivation [24]. It is known that interventions planned to educate and motivate adults to independently increase their PA behaviours are effective in changing PA behaviours and, therefore, in improving health status [20]. In this regard, a recent meta-analysis [25] showed that PA interventions that used motivational tools significantly improved CRF with an effect size of 0.48 (95% confidence interval (CI) 0.37–0.60; *p* < 0.001) for two groups, treatment (i.e., PA interventions) versus control. Likewise, in the present study the gamification technique [13] was used as a motivational teaching tool to encourage college student to practice PA and meet the recommendations for the highest possible number of weeks, so that they could obtain significant CRF improvements as a consequence. Whereas in the present study we used a CG to be compared against the IG, it could be interesting for future studies to use two-factor analysis to study health differences between a group following a gamification program and another group following a traditional teaching program but also with a motivation strategy for promoting an active lifestyle.

According to this, gamification has been shown to be a powerful tool to develop behavioural changes that lead to a healthy lifestyle in different contexts, in young and adult populations [24,26]. For instance, a study showed that young adult users of a gamification-based application (app) significantly enhanced physical activities compared with themselves when they exercised alone by up to 15% [27]. In another study in which a gaming electronic tool was used as an interactive PA intervention, a significant difference was found between the time in minutes of PA performed by adults belonging to the IG (i.e., use of gaming tool) with respect to the CG [28].

This approach has been tested for other health related behaviours, such as diet, in younger population. In children, a study showed significant differences in the rates of Mediterranean diet quality measured by the Mediterranean Diet Quality Index (KIDMED) test between the control and experimental groups (*t* = 3.657; *p* ≤ 0.05) after the application of a game-based physical and digital activities program [24]. In another sample of adolescents, those who played a card game called “Fighting for my health” in recesses showed significant improvements pre-post in 5 of 6 nutritional patterns (all *p* < 0.050) in comparison with the CG [29].

All the previously mentioned findings together with the well-known significant influence of the media on the acquisition of lifestyle habits were taken into consideration for the present study when establishing gamification and “Matrix” as a powerful educational tool to promote PA. Therefore, the gamification approach as a motivational tool to increase PA practice could explain the significant association found in this study between attendance of gamification-based lectures of the program and the number of weeks that participants met the PA recommendations. 

When activities in an educative context cause satisfaction or happiness, college students feel more motivated before performing any demanded task, pay more attention, and show greater interest in the subject taught [30]. Therefore, when a gamification approach is carried out as a teaching methodology, the main motivation that students have to frequently attend the lectures is the game atmosphere itself [24]. Another factor that explains the high satisfaction with respect to a subject in the university seems to be the implementation of learning activities outside of the academic context as a result of the gamification character of the program implemented (e.g., the escape from “The Sentinels” by running or cycling carried out in the present study) which includes the establishment of roles, scores, levels, and allowed students to progress weekly based on compliance with rules and with the established schedule. As in the present study, the student awaited the weekly feedback and were eager to see their progress, which might motivate and satisfy them. The satisfaction of college students was very high (i.e., 4.9 up to 5 points), which might be indicating that the gamification approach given to lectures and set in “The Matrix Revolutions” film was effective at encouraging students to practice PA and, therefore, improve their cardiovascular health. Therefore, it seems of importance that future physical education teachers acquire methodological techniques that help them mainly to increase the motivation of their future students and at the same time their physical health levels.

The main limitation of the present study was the small sample size and consequent small statistical power. In addition, the quasi-experimental design can be considered itself a limitation. In this sense, the convenience sampling meant that participants belonging to the IG were not randomly selected since they were already part of a specific student group of a subject intervention program. Another limitation lies in the fact that all students participating in this study belong to the field of sport and health as they were students of the degree in Physical Activity and Sports Sciences. Thus, it makes difficult to generalize from the results and make conclusions about the whole young adult population. Another limitation was the lack of information about anthropometric characteristics to be used as control variables in statistical analyses since they have been shown to be related to fitness outcomes. Also, the fact that PA was not measured using objective instruments is considered a limitation. On the other hand, to the best of our knowledge this is the first study investigating the influence of a gamification-based program as a motivational teaching tool designed to increase PA levels on CRF of college students in a university setting. The application of gamification in a natural teaching university context is also itself a strength.

## 5. Conclusions

The findings of the present study suggest that a gamification-based teaching program designed to increase PA levels in college students has a significant effect on their CRF in comparison with peers of a CG. Furthermore, significant differences were found between groups attending the gamification-based lectures of the subject and groups meeting PA recommendations in college students of IG. There were also significant differences between the group meeting PA recommendations 100% of weeks and the group that did not meet them for CRF improvements. Our results together with those from previous studies suggest that motivating strategies to increase PA levels such as gamification can lead to physical health benefits in youths. Educational institutions in the university context should target and promote PA throughout innovative strategies in order to lead young adults to improve their immediate and longer-term physical health.

## Figures and Tables

**Table 1:** Main missions, objectives and contents of the 5 phases of the “Matrix rEFvolution”.

PHASE	Mission	Objectives and Contents of the Subject Covered
**Recruitment**	The student must demonstrate that he/she has the potential to become one of “The Chosen” to lead “The Resistance” against Matrix.	Favor the creativity and the command of the ICTs. Digital identity and personal brand.
**Rehabilitation**	Be aware of the lies of Matrix (i.e., the corrupt educative system). Unlearn and show predisposition to a more critical learning.	To promote critical awareness. (Physical) Education: pass, present and future.
**Training**	To improve their learning by “fighting” among themselves.	To program Physical Education into their studies. Active methodologies in Education. Shared and formative evaluation.
**Incursion in Matrix**	The student must demonstrate what he/she has learned at this point, trying to get the highest repercussion in both his/her own setting and among the rest of the professors.	To demonstrate their compromise with their learning and with the profession.
**Final Mission**	The students must present their software (i.e., their own innovation project). To locate the CPU of Matrix and insert there the device (software) to restart the system.	To design innovation projects in Physical Education.

ICTs = Information and Communication Technologies. CPU = Central Processing Unit.

**Table 2:** Baseline characteristics of the college students.

	All (n = 112)	CG (n = 56)	IG (n = 56)	*p* ^a^
Age	20.9 ± 1.3	20.8 ± 1.2	21.0 ± 1.4	0.379
Stages completed in 20mSRT	6.5 ± 1.8	6.4 ± 1.7	6.6 ± 1.8	0.523
Estimated VO2max from 20mSRT ^b^	40.0 ± 5.3	39.7 ± 5.2	40.3 ± 5.4	0.523

Values are means ± standard deviation (SD). CG = control group; IG = intervention group; 20mSRT = 20-meter shuttle-run test. *p* ^a^ -value for comparison between groups by one-way analysis of variance (ANOVA). ^b^ Estimated maximal oxygen consumption (VO2max) was calculated using Léger’s equation for adults [15].

**Table 3:** Frequency of attendance to gamification-based lectures of the program, frequency of weeks meeting PA recommendation, and grade of satisfaction with respect to the “Matrix rEFvolution” program of college students from intervention group.

	Intervention Group (n = 56)
Attendance (grade of implication)	
Attendance to <42 lectures	15 (26.8%)
Attendance to 42 lectures	41 (73.2%)
Weeks of PA recommendations	
PA recommendations <15 weeks	16 (28.6%)
PA recommendation 15 weeks	40 (71.4%)
Grade of satisfaction (Mean ± SD)	4.9 ± 0.3

Values are number of cases (%) unless otherwise indicated. SD = standard deviation. 42 lectures represent 100% of attendance. 15 weeks represent 100% of number of weeks meeting PA recommendations.

**Table 4:** Changes in cardiorespiratory fitness for control group and intervention group.

	Pre-intervention	Post-intervention	Difference (Post-Pre)	*p* ^b^
Stages completed in 20mSRT				
CG	6.4 ± 1.7	6.5 ± 1.7	0.1 ± 0.8	0.151
IG	6.6 ± 1.8	8.2 ± 1.9	1.6 ± 1.0	<0.001
Difference (IC-CG)	0.2 ± 2.1	1.7 ± 2.3		
P ^a^	0.213	<0.001		
Estimated VO2max (ml/kg/min) in 20mSRT				
CG	39.7 ± 5.2	40.1 ± 5.2	0.4 ± 2.5	0.213
IG	40.3 ± 5.4	45.1 ± 5.6	4.8 ± 3.0	<0.001
Difference (IC-CG)	0.6 ± 2.5	5.0 ± 7.3		
*p* ^a^	0.151	<0.001		

Values are means ± standard deviation (SD). CG = control group; IG = intervention group. *p* ^a^ -value (read in vertical) for comparison between groups by one-way analysis of covariance (ANCOVA) adjusted by age and sex. *p* ^b^ -value (read in horizontal) for comparison within groups by paired *t*-test. *p* significant level was set at *p* < 0.050.
